# Nanostructured Electrochemical Biosensor for Highly Sensitive Copeptin Detection as a Cardiac Biomarker

**DOI:** 10.1021/acsomega.6c02983

**Published:** 2026-09-10

**Authors:** Duygu Nuhoğlu, Gunseli Guney Kekec, Basak Senturk, Kevser Buse Karadeniz, Eylul Ece Islek Camadan, Afranur Pendar, Yunus Zorlu, Ahmet Can Timucin, Ozge Can, Tanil Kocagoz, Cihat Taşaltın, Esra Zayim, Ilke Gurol

**Affiliations:** † Department of Physics Engineering, Istanbul Technical University, Maslak, Istanbul 34469, Türkiye; ‡ TUBITAK, Marmara Research Center, Materials Technologies, P.O. Box 21, Gebze, Kocaeli 41470, Türkiye; § Department of Chemistry, Faculty of Arts and Science, Yildiz Technical University, Istanbul 34220, Türkiye; ∥ Department of Medical Biotechnology, Graduate School of Health Sciences, 162328 Acibadem Mehmet Ali Aydinlar University, Istanbul 34752, Türkiye; ⊥ Department of Chemistry, 52962 Gebze Technical University, Gebze, Kocaeli 41400, Türkiye; # Department of Molecular Biology and Genetics, Faculty of Engineering and Natural Sciences, Acibadem Mehmet Ali Aydinlar University, Istanbul 34752, Türkiye; ∇ Department of Molecular Biology and Genetics, Graduate School of Natural and Applied Sciences, Acibadem Mehmet Ali Aydinlar University, Istanbul 34752, Türkiye; ○ Department of Biomedical Engineering, Faculty of Engineering and Natural Sciences, Acibadem Mehmet Ali Aydinlar University, Istanbul 34752, Türkiye; ◆ Department of Biomedical Engineering, Graduate School of Natural and Applied Sciences, Acibadem Mehmet Ali Aydinlar University, Istanbul 34752, Türkiye; ¶ Department of Medical Microbiology, School of Medicine, Acibadem Mehmet Ali Aydinlar University, Istanbul 34752, Türkiye

## Abstract

Copeptin is an important biomarker for the diagnosis of acute myocardial infarction in the troponin-blind period. In this study, a peptide-based nanostructured electrochemical biosensor was developed on a screen-printed electrode platform, enabling a disposable sensing strategy suitable for on-site applications. A copeptin-specific peptide, synthesized for this study, was employed as the biorecognition element to provide binding sites for the target. The UiO-66-NH_2_ metal–organic framework was synthesized to improve the electrochemical response and served as a functional interface due to its high surface area. The proposed biosensor platform provides a sensitive detection of copeptin with a limit of detection of 228.5 pg/mL, offering a promising low-cost and efficient peptide-based electrochemical sensing platform.

## Graphical abstract

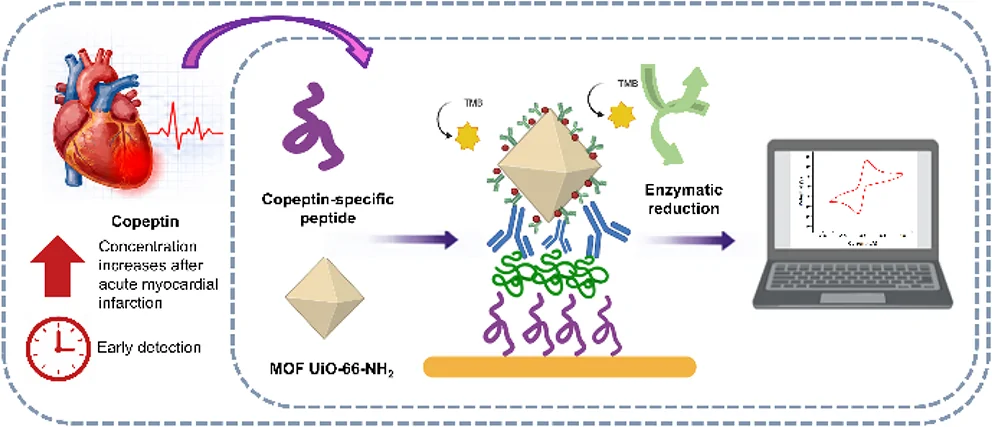

## 1 Introduction

Cardiovascular diseases (CVDs) are a group of disorders affecting the heart and blood vessels and are the leading cause of mortality worldwide. According to the World Health Organization’s statistical data, an estimated 19.8 million people died from CVDs in 2022, indicating approximately 32% of all global deaths.^1^ Among CVDs, acute myocardial infarction (AMI) is a major cause of morbidity and fatality. Therefore, accurate and timely diagnosis of AMI is vital, and the use of specific biomarkers, whose concentrations differ from those in healthy individuals, plays a crucial role in the detection process. Cardiac troponins (cTnI^2^ and cTnT) are among the most extensively studied biomarkers due to their well-established clinical relevance^3^ and are considered the gold-standard biomarkers for the diagnosis of cardiac events because of their high specificity and sensitivity. They significantly increase in the bloodstream at 6–9 h after myocardial injury, peaking at 12–48 h.^4^ Although cardiac troponins are considered gold-standard biomarkers, the delay of the release and elevation of troponins in the initial hours, the so-called “troponin-blind period”,^5^ make cTnI and cTnT unsatisfactory for AMI diagnosis within the first 2 h of the onset of symptoms. The availability of a biomarker that reveals earlier stages of ischemia could significantly improve diagnostic accuracy and clinical decision-making in the initial stages of AMI. Especially copeptin, a novel biomarker whose concentration increases in the blood within 2 h and reaches a peak at 6 h, is a promising candidate for early diagnosis.^6^ Moreover, the combined use of copeptin with troponins^7^ has been reported to enable early diagnosis of AMI,^8^ emphasizing the need for rapid and sensitive copeptin detection platforms.

Copeptin, a 39-amino-acid-long glycopeptide, is a peptide that forms the C-terminal portion of the arginine vasopressin (AVP) precursor, which regulates the body’s sodium–water balance, and is formed by splitting from this precursor.^9^ AVP in circulation significantly limits its clinical applications and direct measurement because of its relatively short half-life. Whereas copeptin is highly stable even at room temperature, making it an indirect but reliable indicator of biological activities such as response to endogenous stress^7^ and ischemic stroke.^10^ Further, copeptin has a prognostic value in the early stage of AMI; Maisel et al. reported that the copeptin concentration increases above 14 pmol/L (≈160 pg/mL) within 2 h after symptoms and could be associated with the outcomes of AMI.^6^ Consequently, accurate and specific detection of copeptin can contribute to the early diagnosis of AMI and to reducing the risk of AMI-related mortality.

Analytical platforms developed for copeptin determination are commonly based on the chemiluminescence (CL) technique.^9^ While these methods provide high sensitivity and low detection limits in biomarker analysis,^11^ their portability is limited by the need for complex and expensive equipment such as optoelectronic infrastructure. Although these approaches offer high sensitivity, they generally require specialized infrastructure and a laboratory environment. In contrast, screen-printed electrode (SPE)-based electrochemical biosensor platforms are advantageous for integration into portable systems due to their disposable design and small sample volume requirements.^12^ SPE platforms, based on electrochemical measurement principles, offer promising alternatives for the rapid and on-site (point-of-care) detection of biomarkers. This situation points to a significant gap in the development of low-cost, user-friendly diagnostic systems that allow on-site analysis without requiring expert personnel. In this context, biorecognition elements play a critical role in determining the sensitivity and performance of biosensors. While antibodies are the most commonly used recognition elements, peptide-based systems have recently gained attention for their higher stability, ease of synthesis, and tunable binding affinity.^13^ However, peptide-based recognition strategies for copeptin detection remain largely unexplored. Accordingly, the performance of electrochemical biosensors is directly related to the efficiency of interfacial biorecognition events and the design of the sensing architecture. In multi-layered sensing architectures involving peptide-based bioreceptors, metal–organic framework (MOF) nanostructures, and enzyme-labeled antibodies, the surface chemistry and the interactions among these components play a critical role in determining sensor sensitivity. Therefore, optimizing each layer of the sensor individually is critical as the surface architecture formed by these components can dramatically influence the overall electrochemical response of the sensor, and the effects of different concentrations on binding kinetics, steric hindrance, and enzymatic signal generation capacity can be clearly observed in the electrochemical signals.

In this study, we report a novel biosensor for the detection of copeptin by using an electrochemical sensor platform with a specifically synthesized copeptin-specific peptide as a biorecognition element and MOF UiO-66-NH_2_ serving as an amino-functionalized nanostructure for antibody immobilization and functioning as a signal amplifier. In the developed copeptin biosensor, due to the complex nature of biomolecular interactions, it was deemed necessary to individually optimize the concentrations of the four main components (peptide bioreceptor, MOF UiO-66-NH_2_, HRP-labeled secondary antibody that generates a signal by enzymatically reacting with TMB, and target analyte, copeptin antigen). Such optimization not only determines the conditions under which the sensor provides a maximum response but also contributes to extending the linear operating range of the system and creating a more stable biological architecture on the surface. The enzymatic amplification mechanism, which is particularly critical in electrochemical measurements, combined with the high surface area and porosity properties of the MOF structure, can lead to problems such as signal saturation, fluctuation, or insufficient catalytic activity if proper optimization is not performed. Therefore, multiple concentration ranges were tested for each component, and the optimal values yielding the highest electrochemical responses were determined. In this context, the proposed sensor platform provided sensitive detection of copeptin, highlighting its potential applications in the early diagnosis of AMI. Notably, this study presents the first report of a peptide-based electrochemical biosensor for copeptin antigen detection using a synthesized copeptin-specific peptide as the biorecognition element and introducing a unique immobilization strategy. In addition, MOF UiO-66-NH_2_ was employed for the first time in copeptin detection as a signal-enhancing nanomaterial, offering a novel MOF UiO-66-NH_2_-based approach to electrochemical biosensing.

## 2 Experimental Methods

### 2.1 Chemicals and Apparatus

Ethanolamine, 11-mercaptoundecanoic acid, 6-mercaptohexanol, bovine serum albumin (BSA), 3,3′,5,5′-tetramethylbenzidine (TMB), *N*,*N*′-diisopropylcarbodiimide (DIC), trifluoroacetic acid (TFA), triisopropylsilane (TIS), and 2,2′-ethylenedioxy)­diethanethiol (EDT) were purchased from Sigma–Aldrich. 1-(3-(Dimethylamino)­propyl)-3-ethylcarbodiimide hydrochloride (EDC) was acquired from TCI. *N*-Hydroxysuccinimide (NHS) and 2-aminoterephthalic (H_2_BDC-NH_2_) from Aldrich. l-Form amino acids, Rink Amide resin, Oxyma, and the Razor device were obtained from CEM Corporation, USA. The recombinant rabbit monoclonal antibody against copeptin (capture antibody) was purchased from Invitrogen. Copeptin (human) antigen was obtained from Phoenix Pharmaceutical. Goat anti-rabbit IgG (H+L) secondary antibody and HRP (HRP-linked secondary antibody) were purchased from Invitrogen. Potassium ferricyanide K_3_[Fe­(CN)_6_], potassium ferrocyanide K_4_[Fe­(CN)_6_], and phosphate-buffered saline (PBS) were obtained from Sigma, and zirconium­(IV) chloride (ZrCl_4_) was purchased from ABCR.

Solid-phase peptide synthesizer (Liberty Blue, CEM Corporation, USA) was used for peptide synthesis. The structural and morphological characterization of the synthesized MOF UiO-66-NH_2_ was performed by using powder X-ray diffraction (PXRD) and scanning electron microscopy (SEM). Electrochemical sensor experiments were carried out using a custom-built electrochemical measurement system (EBTRO potentiostat) with a single-input standard electrode cell stand. An SPE with a 3-mm-diameter working electrode was used, with gold serving as the working, reference, and counter electrodes.

### 2.2 MOF UiO-66-NH_2_ Synthesis

UiO-66-NH_2_ was synthesized according to a previously reported procedure with slight modifications.^14^ In a typical synthesis, 40 mg of ZrCl_4_ and 31 mg of H_2_BDC-NH_2_ were dissolved in 10 mL of DMF. The mixture was sonicated for 15 min to ensure complete dissolution of the precursors. Subsequently, 0.3 mL of acetic acid was added, and the mixture was further sonicated for 5 min. The resulting homogeneous mixture was then transferred into a Teflon-lined stainless-steel autoclave and heated at 120 °C for 24 h under solvothermal conditions. After the reaction, the autoclave was allowed to cool naturally to room temperature. The obtained light-yellow precipitate was collected by centrifugation, washed three times with DMF and ethanol to remove residual reagents and solvent molecules, and finally dried at 100 °C overnight.

### 2.3 Peptide Design, Synthesis, and Binding Affinity Measurement

As copeptin lacks an experimentally resolved structure, peptide prediction was performed computationally using a 500 ns molecular dynamics simulation of the full preprohormone arginine vasopressin structure obtained from AlphaFold (AF-P01185-F1). Following the simulation, conformational stability was assessed using root-mean-square deviation (RMSD) analysis, and regions of low root-mean-square fluctuations (RMSF) interacting with the copeptin sequence of arginine vasopressin were identified as structurally rigid and likely to engage with copeptin. These low-fluctuating regions were subsequently cleaved into 30-amino-acid fragments to generate candidate inhibitory peptides potentially targeting copeptin. *In vitro* utilized peptide was then selected among candidate peptides using docking to the folded copeptin structure, followed by molecular dynamics simulations for 500 ns with multiple replicates and estimation of relative binding free energy via the molecular mechanics Poisson–Boltzmann surface area solvation (MM-PBSA) method.

Peptide synthesis was performed according to the method previously described by the authors of the reference study.^15^ Briefly, peptides were synthesized using a solid-phase peptide synthesizer following the Fmoc (9-fluorenylmethoxycarbonyl) protocol. l-Form amino acids and 0.2 M Rink Amide resin (0.7 mmol/g loading) were used to obtain C-terminally amidated peptides, and synthesis was carried out on a 0.10 mmol scale. The resin was first swollen in DMF for 30 min before synthesis. Fmoc deprotection was performed using 20% piperidine in DMF, and amino acid coupling was achieved using 0.5 M DIC as the coupling reagent and 1.0 M Oxyma as the activator. After completion of the synthesis, peptides were cleaved from the resin using a cleavage cocktail containing TFA, TIS, EDT, and deionized water for 30 min at 38 °C using a Razor device. The cleaved peptides were precipitated with cold diethyl ether, centrifuged at 4000 rpm for 3 min, washed three times, and air-dried overnight.

Recombinant copeptin (Cloud-Clone Corp.) was used to assess the interaction between copeptin and the copeptin-specific peptide via MicroScale Thermophoresis (MST; Monolith, NanoTemper Technologies). Prior to analysis, the protein solution was buffer-exchanged into 1× PBS and labeled at 10 μM using the Red NHS Protein Labeling Kit (NanoTemper Technologies), following the manufacturer’s protocol. 20 nM of the labeled protein was used for the binding experiments. The copeptin-specific peptide was serially diluted 16 times for the binding experiments. MST measurements were carried out according to standard pretest, binding check, and affinity protocols using the MO. Control and Affinity Analysis software. PBS supplemented with 0.05% Tween-20 served as the assay buffer, and Monolith Premium Capillaries were used to reduce aggregation. Samples were incubated for 30 min prior to affinity measurements. During measurements, 50% excitation power and Pico-RED excitation settings were applied.

### 2.4 Preparation of Electrode Surface

#### 2.4.1 Sensor Surface Modification

Bare sensors were measured with 1 M potassium ferricyanide K_3_[Fe­(CN)_6_]/KCl between ±1 V potential range using cyclic voltammetry (CV) to establish a baseline. The electrochemical sensor surface was rinsed sequentially with ethanol and distilled water, then allowed to air-dry. Oxygen plasma treatment was applied to remove organic contaminants and to prepare the gold working electrode area for the subsequent self-assembled monolayer (SAM) formation step by increasing its surface energy and rendering it more hydrophilic. In order to create a SAM surface, sensors were incubated in a 7:3 ratio of 11-mercaptoundecanoic acid and 6-mercaptohexanol solution for 72 h, then washed with distilled water, and the CV measurement using 1 M potassium ferricyanide was repeated to confirm successful SAM modification. For SAM-constructed sensor surfaces, the assay protocol illustrated in Figure 1 was followed.

**Figure 1:**
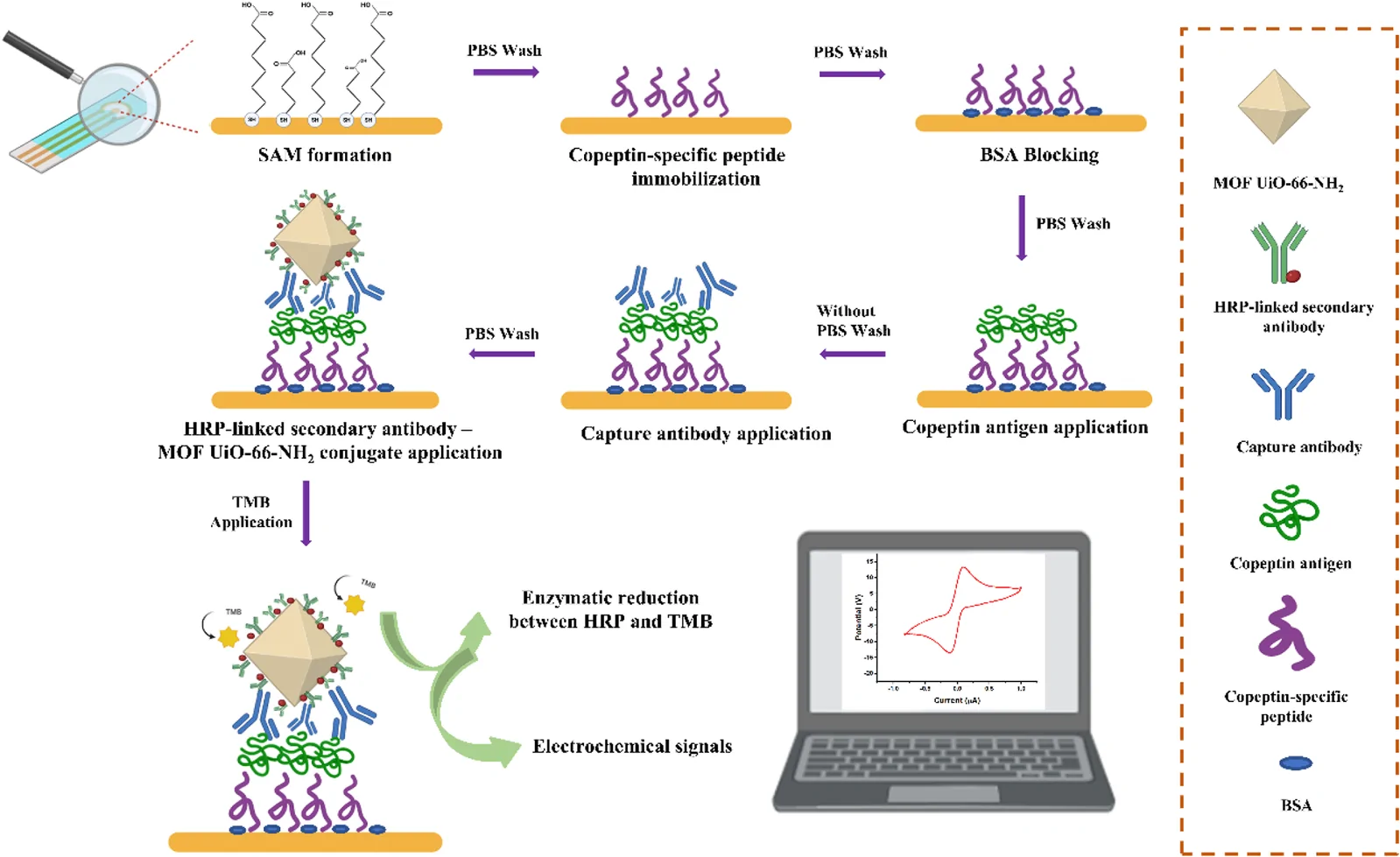
Schematic view of the assay protocol used for electrochemical sensing of copeptin.

Briefly, the SAM-modified sensor surfaces were activated using a 1:1 mixture of 0.4 M EDC and 0.1 M NHS as cross-linking agents and incubated for 1 h at 4 °C to facilitate efficient covalent coupling. After incubation, the surface was washed with PBS (0.01 M in distilled water), and the copeptin-specific peptide was immobilized to the surface and incubated for 24 h at 4 °C, followed by another PBS wash. Afterward, the covalently immobilized peptides were blocked to eliminate nonspecific interactions in the next steps with BSA (1 mg/10 mL) for 10 min. Then, the copeptin antigen was introduced onto the surface and incubated for 10 min to enable its interaction with the immobilized peptide via noncovalent binding. At this step, the sensor surface was not washed; the capture antibody was applied and allowed to adsorb for 10 min and washed with PBS. HRP-linked secondary antibody and MOF UiO-66-NH_2_ conjugate structure was prepared as follows: dispersed and EDC-NHS treated MOF UiO-66-NH_2_ was incubated with the prepared HRP-linked secondary antibody solution. Thereafter, the conjugate was applied to the sensor surface, incubated for 15 min at room temperature, and washed with PBS. To initiate the enzymatic reaction, TMB was added to the sensors and measured for electrochemical characterization. In this sensor structure, SAM formation provided a functionalized interface for the stable covalent immobilization of the copeptin-specific peptide, serving as the biological recognition element. The capture and HRP-linked secondary antibodies formed a sandwich-like structure, enhancing specificity against the copeptin antigen. The MOF UiO-66-NH_2_ acted as a high-surface-area carrier to increase HRP loading, enabling efficient enzymatic reaction with TMB and amplifying the electrochemical response.

Optimization experiments were conducted to determine the optimal concentrations of each component, and the results are presented in Table 1.

**Table 1:** A Series of Optimization Experiments

	Copeptin-specific peptide (mg/mL)	MOF UiO-66-NH_2_ (mg)	Secondary antibody (mg/mL)	Copeptin antigen (mg/mL)	Capture antibody (mg/mL)
Copeptin-specific peptide opt.	0.01, 0.02, 0.03, 0.04, 0.05	0.375	0.02	0.001	0.5
MOF UiO-66-NH_2_ opt.	0.04	0.35, 0.375, 0.4	0.02	0.001	0.5
Seconder antibodyopt.	0.04	0.375	0.02, 0.025, 0.03, 0.035	0.001	0.5
Copeptin antigen opt.	0.04	0.375	0.035	0.0001, 0.0005, 0.001, 0.0015, 0.002	0.5

First, copeptin-specific peptide optimization was performed by testing a range of concentrations (0.01, 0.02, 0.03, 0.04, and 0.05 mg/mL). The operating range for the peptide was determined on the basis of theoretical surface coating calculations considering peptide sizes and the active surface area of the sensor. Based on preliminary studies, the amounts of MOF UiO-66-NH_2_ (0.375 mg), the capture antibody concentration (0.02 mg/mL), and the copeptin antigen concentration (0.01 mg/mL) were kept constant. The concentration range at which the sensor can provide a linear response was selected by considering the maximum capacity of binding events expected to occur on the sensor’s surface. On the basis of cyclic voltammetry investigations, 0.04 mg/mL was selected as the optimal peptide concentration and used in the subsequent steps. After that, the amount of MOF UiO-66-NH_2_ was tested using 0.35, 0.375, and 0.4 mg. The best analytical performance was obtained at 0.375 mg. Next, the concentration of the HRP-linked secondary antibody was optimized, and 0.035 mg/mL was determined for subsequent investigations. At the final step of the procedure, copeptin antigen concentrations ranging from 0.0001 to 0.002 mg/mL were tested to determine the response profile of this structure, and the limit of detection (LOD) was calculated.

#### 2.4.2 Measurements

The main criterion used was the change in the oxidation peak current obtained from the system when the concentration of the relevant component was increased; the concentration that produced the highest and most stable peak current was selected as the starting condition for the next stage. As a result, the sensor architecture was optimized step by step to determine the most efficient operating conditions. First, CV measurements were taken to examine the electrochemical behavior of the system in each modification step, and the oxidation peak currents generated at different concentration levels were compared. The CV results were used as the basic criterion to evaluate the effectiveness of the biological architecture created by the sensor components on the surface. Subsequently, amperometric measurements were performed to verify the dynamic response and current-generation capacity of the sensor at the determined optimal concentrations; the measurements were performed at the potential value where the maximum peak current was observed. As a result, both the voltammetric and amperometric performances of the sensor were evaluated comprehensively, and the most suitable operating conditions were determined. Also, electrochemical impedance spectroscopy (EIS) was employed to evaluate the interfacial charge-transfer resistance changes upon antigen binding. This technique provides high sensitivity for detecting low antigen concentrations and allows monitoring of the biosensor surface modifications. Subsequently, EIS measurements were performed using 5 mM [Fe­(CN)_6_]^3–/4–^ in PBS solution as the redox probe to evaluate the sensor response at antigen concentrations of 0.0001, 0.0005, and 0.001 mg/mL.

## 3 Results and Discussion

### 3.1 Binding Affinity between Copeptin-Specific Peptide and Copeptin Antigen

MST experiments were conducted to determine the binding affinity between the copeptin-specific peptide and the recombinant copeptin. MST analysis revealed that the copeptin-specific peptide binds to its target with a dissociation constant (*K*_d_) of 218 nM, indicating a relatively high-affinity interaction (Figure 2). Experimental results confirm that the in silico peptide design successfully yielded a high-affinity peptide, supporting the validity of the computational prediction approach.

**Figure 2:**
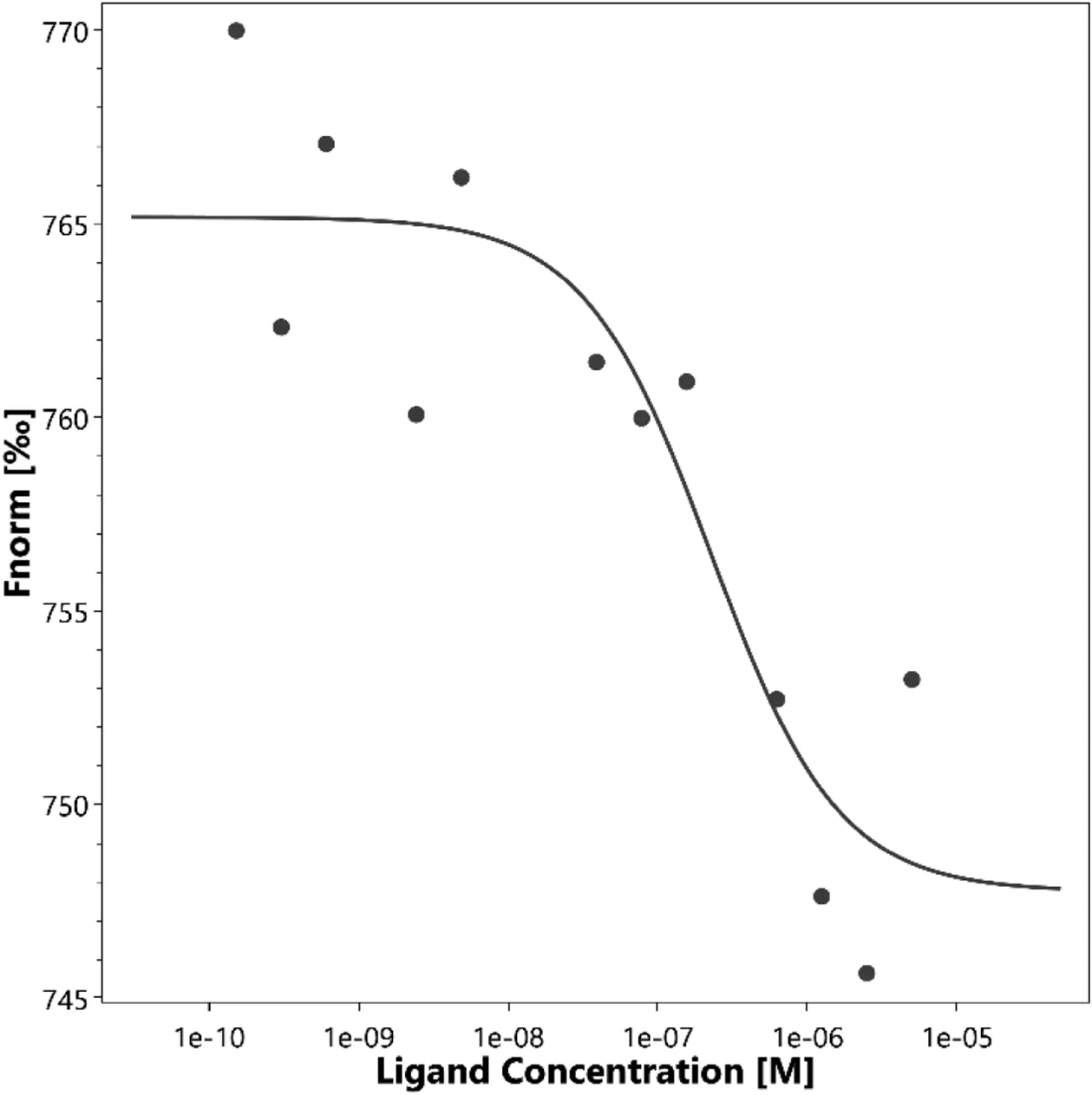
Dose–response curve of copeptin-specific peptide.

### 3.2 MOF UiO-66-NH_2_ Characterization

The PXRD pattern of the synthesized MOF UiO-66-NH_2_ is shown in Figure 3a. The experimental diffraction pattern exhibits good agreement with the simulated pattern, indicating the formation of the framework. Characteristic diffraction peaks observed at 2θ = 7.4°, 8.5°, and 25.7° correspond to the (111), (200), and (400) crystal planes, respectively, confirming the successful synthesis of MOF UiO-66-NH_2_.^16^ The morphology and particle size of the MOF were further investigated using SEM and statistical analysis (Figure 3b, 3c). As shown in Figure 3b, the SEM image reveals intergrown nanocrystals forming irregular clusters, which is consistent with previously reported MOF UiO-66-NH_2_ materials.^17,18^ Based on the statistical size distribution analysis shown in Figure 2c, the average particle size was determined to be 368 ± 85 nm. This submicron size, together with the abundant surface amino groups of MOF UiO-66-NH_2_, provides a favorable platform for the conjugation of secondary antibodies, thereby supporting the signal amplification strategy.^19,20^

**Figure 3:**
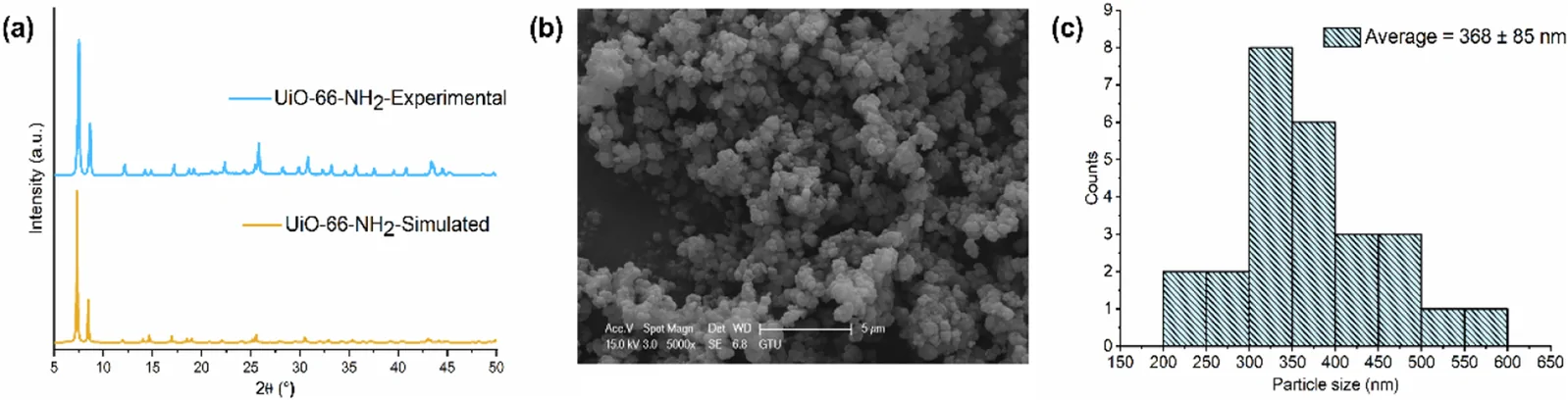
(a) PXRD pattern, (b) SEM image of the synthesized MOF UiO-66-NH_2_, and (c) statistical size distribution analysis.

### 3.3 Cyclic Voltammetry and Amperometry Characterization of Copeptin on SPE

The amount of copeptin-specific peptide immobilized on the sensor surface was optimized in order to achieve the maximum detection performance for the copeptin antigen. This optimization aimed to identify the peptide loading that provides the highest sensing response by ensuring efficient antigen recognition while maintaining favorable electron transfer properties at the sensor interface, with the CV results shown in Figure 4.

**Figure 4:**
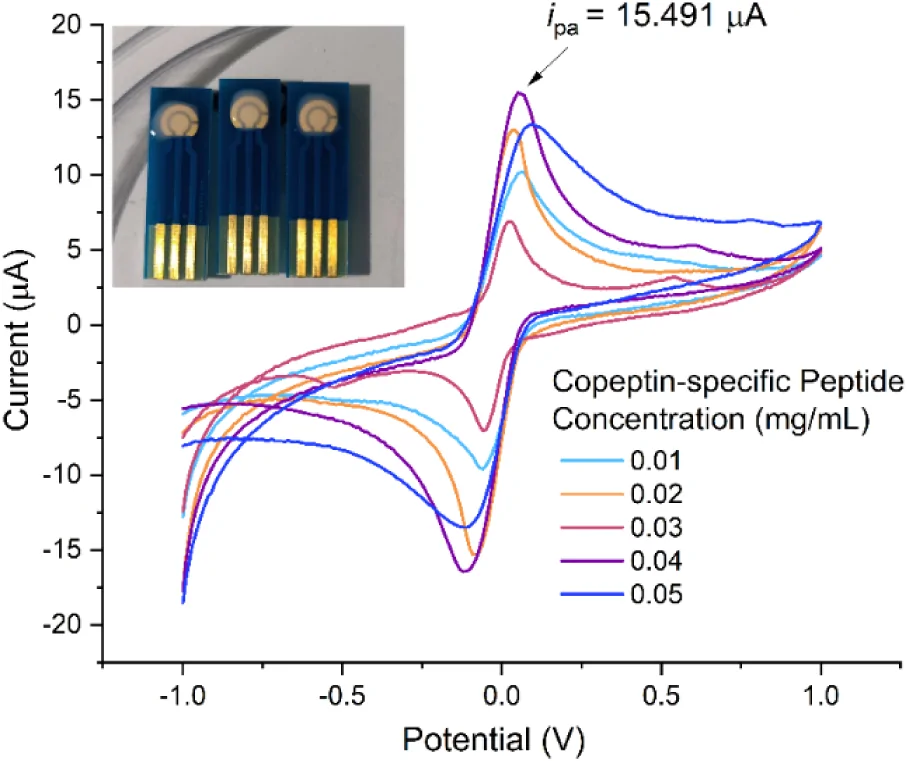
CV curves for copeptin-specific peptide concentrations, inset: Image of the prepared sensors.

The sensor responses were evaluated by immobilizing the copeptin-specific peptide on the electrode surface at different concentrations, as given in Table 1. The concentration of 0.04 mg/mL was selected for the subsequent experiments, as it resulted in the highest peak current of 15.491 μA.

To enhance enzymatic signal generation in CV measurements, an optimization study was performed to determine the optimal amount of high-surface-area MOF UiO-66-NH_2_ for the immobilization of signal-enhancing HRP-labeled antibodies. MOF UiO-66-NH_2_ amounts of 0.35, 0.375, and 0.40 mg were tested to determine the optimal loading. The CV results indicate that the response strongly depends on the amount of MOF UiO-66-NH_2_ immobilized on the working electrode surface, Figure 5a. When 0.35 mg MOF UiO-66-NH_2_ was used, the peak current remained relatively low, which can be attributed to the insufficient active sites for HRP-linked secondary antibody. Increasing the amount to 0.375 mg yielded the highest peak current of 13.567 μA, suggesting more accessible active sites for redox reactions. However, a further increase resulted in a decrease in the peak current due to excessive MOF UiO-66-NH_2_ coverage limited access to active sites. Therefore, 0.375 mg was selected as the optimal amount due to the most stable enzyme immobilization.

**Figure 5:**
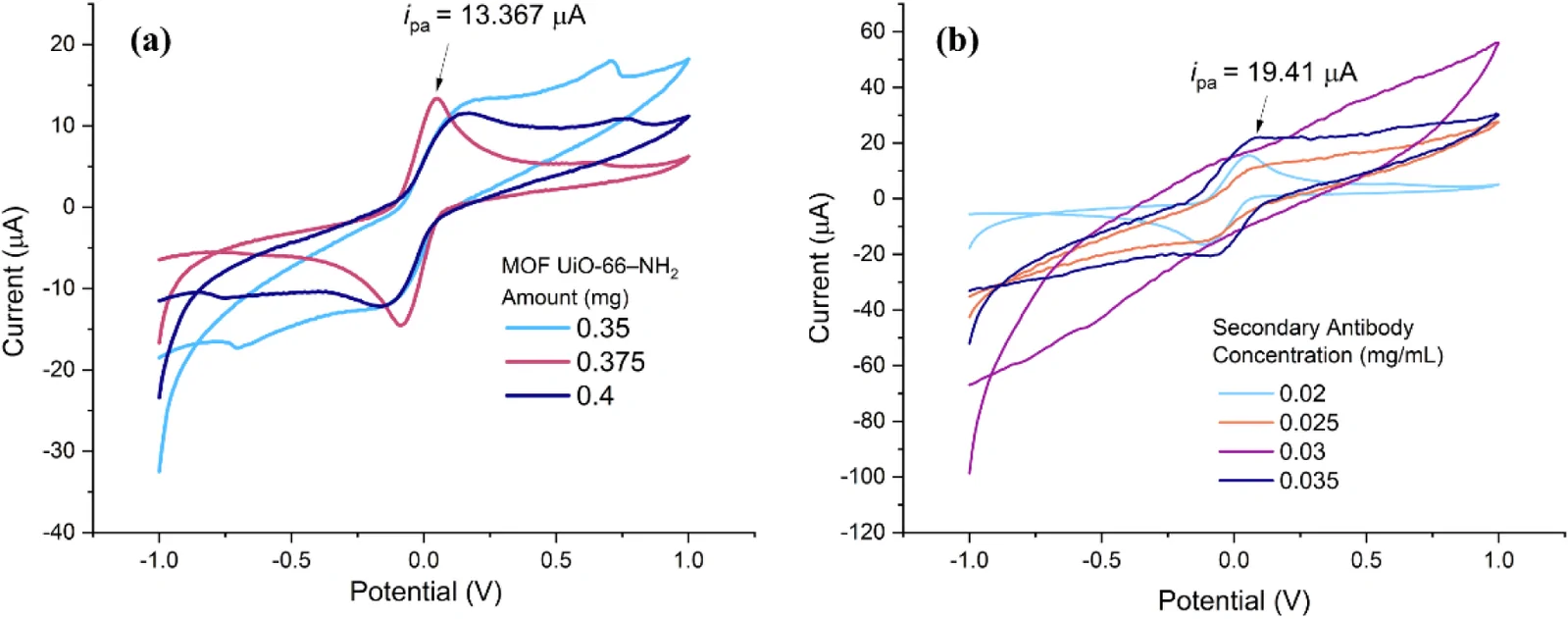
CV curves for (a) different MOF UiO-66-NH_2_ amount, (b) secondary antibody concentrations.

In the final step, the optimal concentration of the enzyme-labeled secondary antibody was determined. Measurements were carried out at different concentrations (0.02, 0.025, 0.03, 0.035 mg/mL), and 0.035 mg/mL was determined to be the optimal concentration (Figure 5b). Furthermore, based on the applied MOF UiO-66-NH_2_ concentration, the stoichiometric ratio of MOF UiO-66-NH_2_ to secondary antibody was estimated to be approximately MOF UiO-66-NH_2_ : Ab ≈ 10.7:1 (w/w).

Using the optimized copeptin-specific peptide and secondary antibody concentrations along with the ideal MOF UiO-66-NH_2_ loading, the analytical range for electrochemical copeptin detection was determined. Finally, the sensor’s response to varying antigen concentrations was used to construct a calibration plot and evaluate the analytical performance of the developed electrochemical platform. This analysis enabled the determination of the linear dynamic range and LOD for the target copeptin antigen, thereby confirming the sensor’s analytical performance and potential for practical application. Copeptin antigen concentrations ranging from 0.0001 to 0.002 mg/mL were evaluated, with the resulting cyclic voltammograms (CVs) presented in Figure 6a. Within this range, both the anodic (*i*_Pa_) and cathodic (*i*_Pc_) peak currents increased linearly with the copeptin concentration, as demonstrated in the inset of Figure 6a.

**Figure 6:**
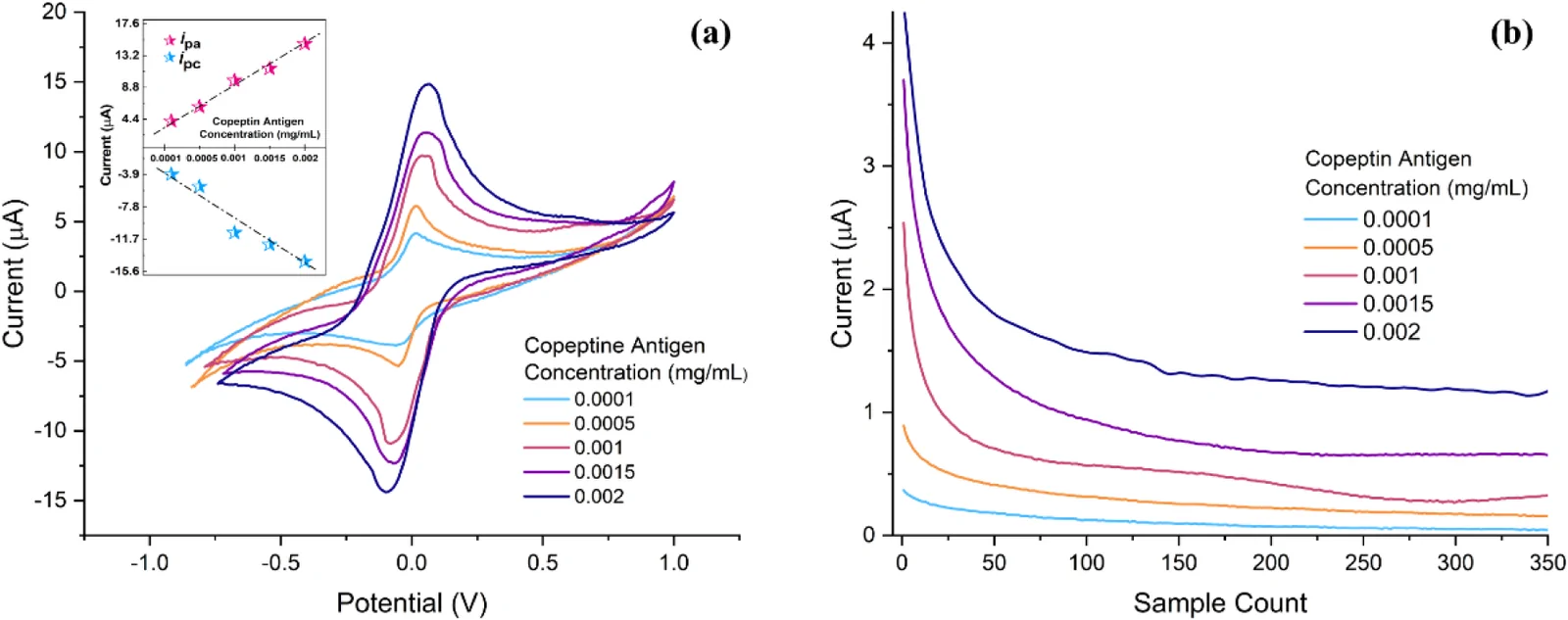
(a) CV curves for copeptin antigen concentrations, inset: corresponding *i*_Pa_ and *i*_Pc_ versus copeptin antigen concentrations, (b) amperometric response for copeptin antigen concentrations.

As shown in Figure 6b, the current decreased overtime for the tested copeptin antigen concentrations and stabilized at a constant value. The amperometric curves confirm that the enzymatic reactions occurring on the sensor surface proceed in a regular and controlled manner, depending on the copeptin antigen concentration.

As illustrated in the calibration plot, the anodic peak currents exhibited a linear dependence on the concentration of the copeptin antigen. The limit of detection (LOD) was calculated following the method described by Ayankojo et al.^21^ The resulting linear regression equation and its corresponding correlation coefficient (*R* ^2^ = 0.99) are presented in Figure 7, demonstrating excellent linearity across the investigated range. The LOD was found to be 228.5 pg/mL.

**Figure 7:**
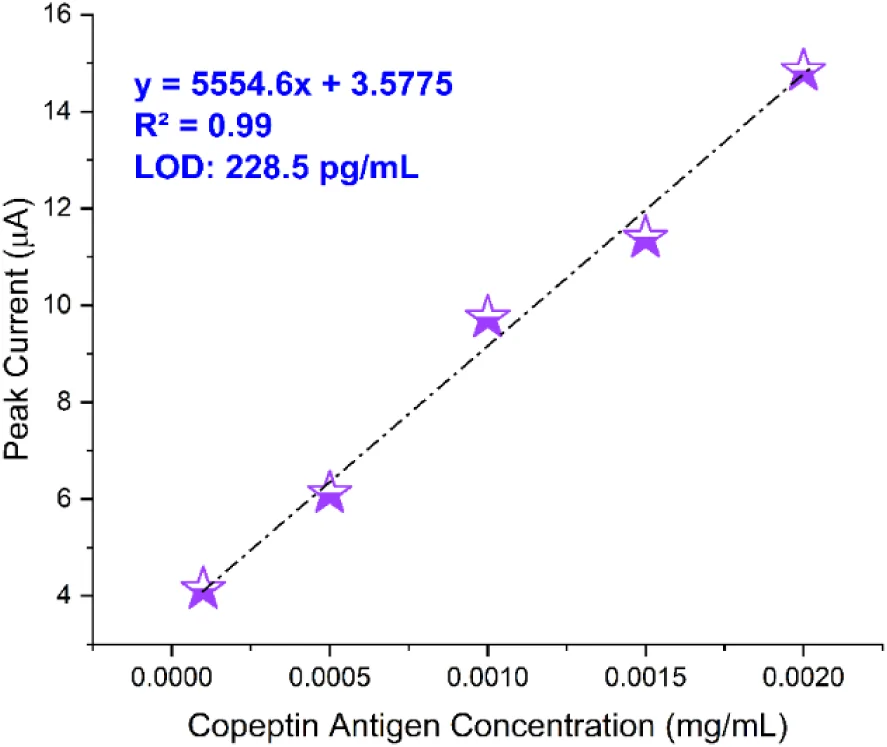
Calibration plot for copeptin antigen concentrations. The dashed line is a linear regression fit.

The sensing performance of the proposed sensor was compared with recently reported sensors, as shown in Table 2. As summarized, several analytical platforms have been reported for the detection of copeptin using different sensing strategies. Most of the previously reported sensing platforms rely on the chemiluminescence (CL) analytical method, which typically provides very low detection limits due to the inherently high sensitivity of luminescence-based signal transduction, where the signal is generated through light emission produced during the reactions. Although this method achieves an LOD significantly lower than those obtained by the electrochemical (EC) sensing method, EC platforms offer important practical advantages such as simplicity, point-of-care detection, and compatibility with portable devices.

**Table 2:** Comparison of the Proposed Electrochemical Biosensor for the Detection of Copeptin

Sensing platform	Analytical method	Linear range	LOD (pg/mL)	Refs
Co^2+^-ABEI-AuNPs-PDA (antibody)	CL	1–10,000 pg/mL	0.325	^9^
Co^2+^-ABEI-Fe_3_O_4_@void@C (antibody)	CL	1–10,000 pg/mL	0.40	^22^
TiO_2_-TCPP-ABEI-based CRET (antibody)	CL	0.005–1 ng/mL	1.54	^23^
BSA/Ab-copeptin/RGO–TiO_2_/ITO (antibody)	EC	10–500 ng/mL	148	^24^
Copeptin-specific peptide	EC	1000–20,000 ng/mL	228.5	This work

The EC analytical method reported in the literature, the antibody-based EC sensor on an ITO electrode surface (BSA/Ab-copeptin/RGO–TiO_2_/ITO)^24^ achieved a LOD slightly below the clinical threshold. In comparison, the present study employs a copeptin-specific peptide as the recognition element on the SPE surface, and the obtained LOD (228.5 pg/mL) remains within the same order of magnitude as other EC sensing methods. Moreover, this study represents a pioneering peptide-based electrochemical platform for copeptin detection integrated with SPE, offering advantages such as disposable electrode formats that minimize cross-contamination and eliminate the need for complex instrumentation, thereby enabling rapid and reproducible measurements.

### 3.4 Electrochemical Impedance Spectroscopy Characterization of Copeptin on SPE

EIS measurements were performed to investigate the interfacial properties of the peptide-modified electrode upon exposure to different concentrations of the copeptin antigen (0.0001, 0.0005, and 0.001 mg/mL). The corresponding Nyquist and Bode plots are presented in Figure 8.

**Figure 8:**
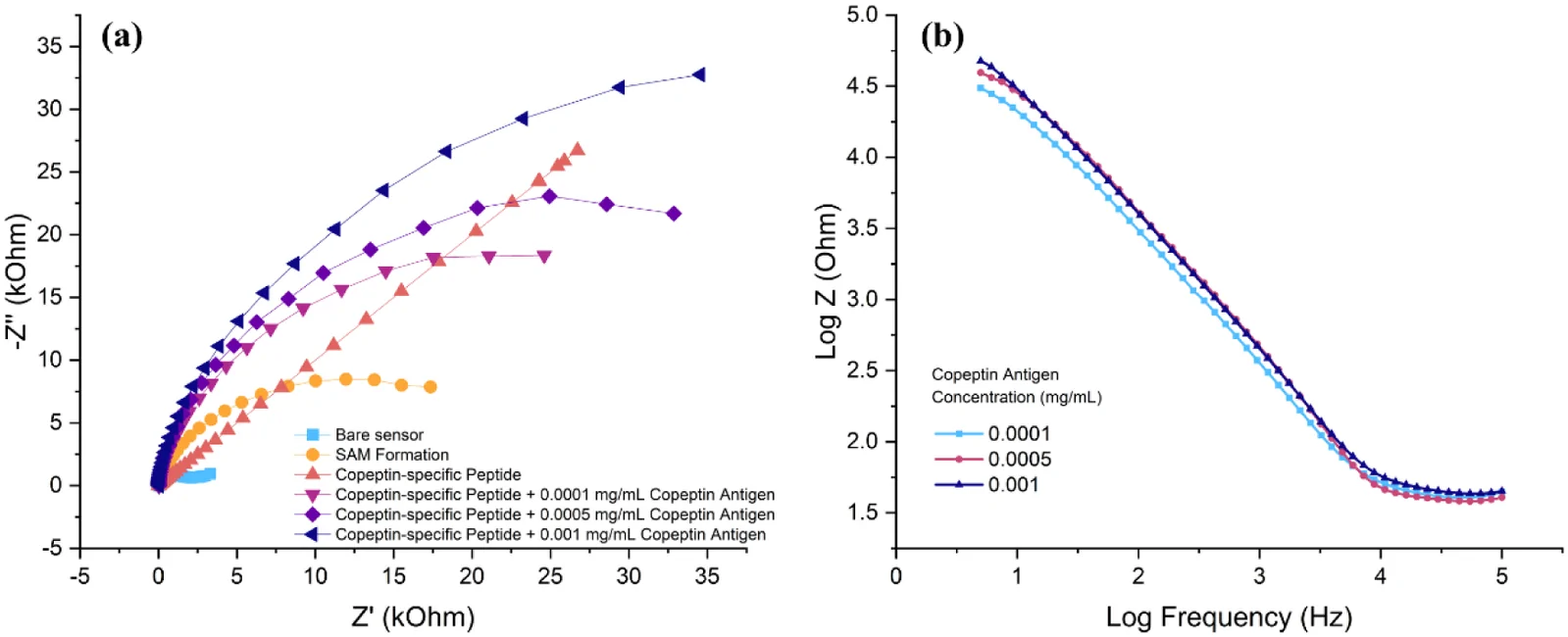
(a) Nyquist plots and (b) Bode magnitude plots of peptide-modified electrodes in the presence of different concentrations of copeptin antigen.

The Nyquist plots provide a representation of the stepwise surface modification and subsequent copeptin antigen-binding processes. As shown in Figure 8a, the bare sensor surface exhibits the smallest semicircle diameter, indicating low charge-transfer resistance (*R*_ct_ = 1.8 kΩ); following SAM formation, a noticeable increase in *R*_ct_ to 23.8 kΩ was observed, suggesting partial blockage of the modified electrode surface due to the SAM formation. Subsequent copeptin-specific peptide immobilization, the Nyquist response exhibits a more pronounced vertical tendency, indicating a transition toward capacitive-dominated behavior. This suggests that the peptide layer forms an insulating barrier on the electrode surface, significantly suppressing faradaic electron transfer between the redox couple and the electrode interface. The increased surface-blocking effect results in hindered charge-transfer kinetics and enhanced interfacial impedance.

Upon copeptin antigen binding, the Nyquist response tends to recover a more defined semicircular shape. This behavior indicates that the formation of the antigen–peptide complex results in a more defined interfacial structure, shifting the impedance response from predominantly capacitive behavior toward charge-transfer-controlled kinetics. A concentration-dependent increase in the semicircle diameter is observed with increasing copeptin levels, corresponding to a rise in *R*_ct_. The observed increase indicates that antigen binding blocks the electrode surface’s active sites and slows electron transfer kinetics. The progressive increase in *R*_ct_ with increasing copeptin concentration indicates successful antigen–peptide binding, which forms an insulating biolayer that hinders electron transfer between the redox probe and the electrode surface. The highest copeptin concentration (0.001 mg/mL) exhibits the highest *R*_ct_, demonstrating the strongest interfacial resistance due to increased biomolecular layer formation.

The absence of a pronounced Warburg diffusion tail suggests that the impedance response is primarily governed by interfacial charge-transfer processes rather than diffusion-controlled processes. Overall, the systematic increase in *R*_ct_ values with an increasing copeptin concentration confirms the successful recognition interaction between the peptide probe and the target antigen. The Bode plots (Figure 8b) further confirm this behavior. The real impedance increases systematically with increasing copeptin concentration in the low-frequency region. In this region, the impedance is dominated by interfacial charge-transfer processes, whereas at higher frequencies, the system behavior is controlled by solution resistance (*R*_s_). This observation suggests that the formation of an insulating biomolecular layer on the surface, which hinders electron transfer between the redox probe and the electrode interface, causes a significant increase in charge-transfer resistance. These results demonstrate the effectiveness of the developed peptide-based biosensing platform for the detection of copeptin.

## 4 Conclusion

This study provides a unique contribution to the literature by enabling, for the first time, the electrochemical detection of copeptin biomarker with a synthesized copeptin-specific peptide immobilized on an SPE. The detection of copeptin was achieved through peptide–antigen interactions using a MOF-based immunosensor creatively designed as a surface-area-enhancing platform. Further, the determination of copeptin was evaluated with CV and CA, supported by EIS analysis. Thus, the electrical properties of the sensor interface involved in the copeptin–peptide interaction were studied in detail, and their importance in the biorecognition process was summarized. Further, this sensor was constructed with a LOD of 228.5 pg/mL. This comprehensive study presents a novel proof-of-concept validation for copeptin detection using standard laboratory samples. The applicability and clinical utility of the proposed biosensor for practical diagnosis remain to be fully evaluated and validated with real clinical samples, which represents a direction for our future research. Overall, this comprehensive study establishes an encouraging biorecognition platform for adapting electrochemical sensors to cardiac biomarker applications.
